# Timely intubation with early prediction of respiratory exacerbation in acute traumatic cervical spinal cord injury

**DOI:** 10.1186/s12873-021-00530-3

**Published:** 2021-11-13

**Authors:** Takafumi Yonemitsu, Azuna Kinoshita, Keiji Nagata, Mika Morishita, Tomoyuki Yamaguchi, Seiya Kato

**Affiliations:** 1 Department of Emergency and Critical Care Medicine, Wakayama Medical University, 811-1, Kimiidera, Wakayama, 641-8509 Japan; 2 High Care Unit, Wakayama Medical University Hospital, 811-1, Kimiidera, Wakayama, 641-8510 Japan; 3 Department of Orthopedic Surgery, Wakayama Medical University, 811-1, Kimiidera, Wakayama, 641-8509 Japan

**Keywords:** Cervical spinal cord injury, Timely intubation, Respiratory exacerbation, Copious airway secretion

## Abstract

**Background:**

Early routine intubation in motor-complete cervical spinal cord injury (CSCI) above the C5 level is a conventional protocol to prevent unexpected respiratory exacerbation (RE). However, in the context of recent advances in multidisciplinary respiratory management, the absolute indication for intubation in patients with CSCI based on initial neurologic assessment is controversial because of the drawbacks of intubation. This study aimed to redetermine the most important predictor of RE following CSCI after admission without routine intubation among patients admitted with motor-complete injury and/or injury above the C5 level to ensure timely intubation.

**Methods:**

We performed a retrospective review of patients with acute traumatic CSCI admitted to our hospital without an initial routine intubation protocol from January 2013 to December 2017. CSCI patients who developed RE (defined as unexpected emergent intubation for respiratory resuscitation) were compared with those who did not. Baseline characteristics and severity of trauma data were collected. Univariate analyses were performed to compare treatment data and clinical outcomes between the two groups. Further, multivariate logistic regression was performed with clinically important independent variables: motor-complete injury, neurologic level above C5, atelectasis, and copious airway secretion (CAS).

**Results:**

Among 58 patients with CSCI, 35 (60.3%) required post-injury intubation and 1 (1.7%) died during hospitalization. Thirteen (22.4%) had RE 3.5 days (mean) post-injury; 3 (37.5%) of eight patients with motor-complete CSCI above C5 developed RE. Eleven of the 27 (40.7%) patients with motor-complete injury and five of the 22 (22.7%) patients with neurologic injury above C5 required emergency intubation at RE. Three of the eight CSCI patients with both risk factors (motor-complete injury above C5) resulted in emergent RE intubation (37.5%). CAS was an independent predictor for RE (odds ratio 7.19, 95% confidence interval 1.48–42.72, *P* = 0.0144) in multivariate analyses.

**Conclusion:**

Timely intubation post-CSCI based on close attention to CAS during the acute 3-day phase may prevent RE and reduce unnecessary invasive airway control even without immediate routine intubation in motor-complete injury above C5.

**Supplementary Information:**

The online version contains supplementary material available at 10.1186/s12873-021-00530-3.

## Background

Patients with acute traumatic cervical spinal cord injury (CSCI) often undergo endotracheal intubation for definitive airway control in cases of acute respiratory exacerbation (refractory desaturation, major dyspnea, and respiratory arrest) induced by pulmonary complications. Prevention of pulmonary complications (copious sputum, atelectasis, pneumonia, and ventilatory disorder) in patients with CSCI by prompt diagnosis and appropriate treatment is necessary to reduce morbidity and mortality [1]. Endotracheal intubation for definitive airway control should ideally be performed under controlled conditions, rather than as an emergency, to avoid deterioration of the neurological status of the patient [2]. Nevertheless, 42% of the patients with CSCI who did not exhibit obvious signs of respiratory impairment in the emergency department (ED) before hospital admission later developed unexpected respiratory exacerbation (RE) and required emergency intubation up to 53 h after admission [3]. Recently, however, there has been remarkable improvement in the nonsurgical care of spinal cord injury, particularly the multidisciplinary care delivered in the pre-hospital, ED, and intensive care unit (ICU) settings [4], thus reducing the need for emergency endotracheal intubation in clinical practice.

Despite advances in the multidisciplinary respiratory management of spinal cord injury, the protocol of absolute indications for routine intubation based on initial neurological assessment has not changed worldwide for nearly two decades. The initial practice of “early routine intubation for CSCI above C5 and complete quadriplegia” has been widely accepted since a retrospective study in 2003 [3]. The 2012 Emergency Neurological Life Support (ENLS) protocol (first version) for traumatic spine injury recommends that all patients with acute complete CSCI above C5, as determined by initial neurological examination, should be intubated as soon as possible before admission (“absolute indication of intubation”) [5]. It is challenging to correctly determine the motor level and type of lesion (complete or incomplete) for physicians providing primary care for patients with CSCI [6], but the latest ENLS protocol (2019, fourth version) does not change the classic absolute indications for early routine intubation in CSCI based on initial neurological assessment [7]. In addition, a previous study in 1998 reported that copious sputum is an independent predictor of the need for mechanical ventilation in patients with CSCI [8]. However, copious sputum is not yet included in the general parameters for urgent intubation in the 2019 ENLS protocol [7].

Adverse events related to mechanical ventilation after intubation in addition to those during intubation should also be taken into consideration in determining the indication for CSCI tracheal intubation. Endotracheal intubation and hypoxia can stimulate an adverse bradycardic response in patients with CSCI [9]. Atelectasis and pneumonia are the leading causes of respiratory failure among these patients [1]. A study in rats reported that mechanical ventilation in spinal cord injury leads to local inflammation in the lung and spinal cord via inflammatory cytotoxic cytokines and critical mediators, without direct tissue injury [10]. In mechanically ventilated patients, the risk of developing pneumonia increases by 1–3% per day of intubation [11]. Patients with CSCI have been reported to have a significantly higher incidence of ventilator-associated pneumonia (VAP) than those with thoracic or lumber spine injuries [12]. Tracheostomy followed by intubation and mechanical ventilation have been reported as risk factors for early mortality in patients with CSCI because of the additional physiological stress and compromised natural immunological barrier between the lung and the outside environment [13].

In the context of modern respiratory management strategies, promoting early tracheostomy as an important component of future CSCI management and reconsidering the indications for endotracheal intubation are both important and complement each other. A recent retrospective study reported that early tracheostomy (≤7 days) in CSCI was associated with lower incidence of VAP and shorter duration of ventilatory management, ICU stay, and hospitalization [14]. On the other hand, tracheostomy itself (both early and late) in CSCI is an independent predictor of ventilator dependence, and there are potential disadvantages inherent in empiric tracheostomy without attempting extubation [12]. Modern invasive airway management in CSCI has accelerated the trend toward early tracheostomy with a minimum of 4 days once intubated [15], but several previous studies have shown that one of the independent predictors of the need for tracheostomy is intubation in the ED [16, 17]. Many previous studies investigating the usefulness of tracheostomy for CSCI have not described the indications for tracheal intubation [12, 14–18], reminding us that early routine tracheal intubation for motor-complete injury above C5 is taken for granted. However, the potential risk that absolute indications for intubation based on neurological assessment may increase unnecessary empirical tracheostomies and the limitations of clinical studies that may not rigorously assess the role of tracheostomy have not been fully elucidated. Lee et al. performed a classification and regression tree (CART) analysis and reported that patients with American Spinal Injury Association (ASIA) Impairment Scale (AIS) grade A definitely need tracheostomy and patients with AIS grades other than A need tracheostomy if intubated in the ED [17]. Besides, once the initial significant spinal shock resolves, incomplete injuries may become unmasked after admission [18]. Patients with CSCI determined to be AIS grade A before admission may actually turn out to be a non-AIS grade A patient requiring no intubation or tracheostomy after admission. In other words, some patients determined to be AIS grade B included in motor-complete injury before admission may not require tracheostomy if classic ED routine intubation is not undertaken. Flanagan et al. reported that early tracheostomy may improve respiratory outcomes in CSCI, but they strictly refrained from ever concluding that early intubation is useful for CSCI patients, as a limitation of their study [19]. While early tracheostomy seems to be an important component of the CSCI management bundle, we believe that the classical indications for CSCI intubation based on neurological assessment need to be reconsidered, in line with advances in modern strategies of respiratory management, in order to avoid increasing unnecessary empirical tracheostomies.

We hypothesized that determining predictors of RE based on modern respiratory management may provide new indications for the timing of intubation, thereby reducing unnecessary invasive airway control in patients with acute traumatic CSCI. Therefore, this study aimed to redetermine the most important predictors of RE following CSCI after admission without routine intubation among patients admitted with motor-complete injury and/or injury above the C5 level, so as to ensure timely intubation.

## Methods

### Study design

We conducted a retrospective case-control study including adult patients with acute traumatic CSCI with or without bone injury, consecutively admitted to a single tertiary emergency medical center in Japan from January 2013 to December 2017. This study was approved by the local institutional review board of the study hospital.

### Patient selection

We screened consecutive patients aged 18–89 years with acute traumatic CSCI, who were admitted to the ED of Wakayama Medical University Hospital, and prospectively reviewed their medical records. Patients were diagnosed with CSCI by the emergency physicians and orthopedic surgeons in the ED upon performing neurological examination and magnetic resonance imaging (MRI) after initial emergency, life-saving medical treatment. Patients with acute, traumatic injuries with lack of motor or sensory functions in the sacral segments, S4–S5, were diagnosed with complete CSCI (defined as motor-complete injury if no motor function below the zone of injury was preserved based on the AIS [version 2003] modified from Frankel grade) [20]. During the study period, the ED physician initially intubated patients with CSCI before hospital admission based on the following three indications alone: airway protection, obvious respiratory/circulatory failure, and emergency preoperative procedure. The indication for cervical spine fixation surgery or tracheostomy was dependent on the experience and judgment of the orthopedic surgeon and attending intensivist without standardization.

Patients were excluded from the study as per the following criteria: central cord syndrome, injury severity score (ISS) > 41, uncertain injury level (such as concomitant severe traumatic brain injury), regular outpatient attendance with an orthopedic of our hospital, and transfer within the first 3 days of hospitalization. RE was defined as unexpected, urgent intubation for respiratory resuscitation or re-intubation within 72 h after planned extubation, including after surgery and at the time of rapid respiratory impairment (refractory desaturation, major dyspnea, and respiratory arrest). Patients who underwent empirical tracheostomy without any extubation attempts following pre-admission intubation were not included in the final statistical analysis to evaluate the independent predictors of RE, because the availability of a definitive sputum suctioning route may have significantly reduced the risk of developing RE and led to selection bias. Finally, the selected patients with CSCI were stratified into those affected by RE (hereafter referred to as RE group) and those who were not (control group).

### Outcome measures

We prospectively collected data on baseline characteristics and the severity of traumatic injury, including age, sex, body mass index, Charlson comorbidity index [21], pulmonary centrilobular emphysema in the apex of the lung on cervical computed tomography (CT), cervical ossification of posterior longitudinal ligament and thoracolumbar diffuse idiopathic skeletal hyperostosis on CT, initial Glasgow Coma Scale score, initial bradycardia (heart rate < 60 beats/min) and initial hypotension (systolic blood pressure < 90 mmHg) in the ED; mechanism of injury (fall or motor vehicle accident), ISS, Abbreviated Injury Scale for the chest, concomitant lung injury (lung contusion, lung laceration defined as traumatic pneumatocele on CT, pneumothorax, or hemothorax), bony thorax injury (rib fracture or sternal fracture), and/or thoracic vertebral fracture; as well as AIS, motor-complete injury, estimated CSCI level, and injury level at and above C5. We defined emphysema in the superior sulcus on the CT scan as a decrease in lung function in heavy smokers [22] because data on smoking history were partially lacking. A board-certified radiologist retrospectively confirmed the key findings of all radiographs, CT, and MRIs to confirm the radiological evidence of baseline and injury characteristics. Regarding the patients’ treatment and clinical course, we collected the following data: intubation before hospitalization, initial admission to the ICU, incidence of copious airway secretion (CAS), atelectasis, pneumonia, cervical spine surgery, and tracheostomy; steroid administration, halo vest immobilization until surgery, length of stay in the ED and hospital, dysphasia and/or ventilatory dependence at discharge, and in-hospital death. CAS was defined as retained tracheobronchial secretions attributable to a respiratory cause (acute refractory desaturation, major dyspnea, and abnormality on auscultation) and required airway (including nasotracheal) suctioning every 2 h (or more, as needed) on each patient’s daily flowsheet as recorded by the nursing staff. Atelectasis was assessed with chest radiography as the loss of lung volume, involving clinical hypoxemia and hypophonesis in the affected lung without symptoms of pulmonary infection, as interpreted by the attending physician. Pneumonia was diagnosed based on radiographic parenchymal inflammatory evidence with clinical acute fever requiring antibiotic administration. Empiric antibiotics were not administered routinely in patients with CSCI, except to prevent surgical site infections. We routinely examined the patients for CAS, atelectasis, and pneumonia during the acute 3-day phase after admission or within 24 h before RE.

The primary outcome was RE after admission in patients with traumatic CSCI. We identified and evaluated the possible risk factors contributing to RE. The secondary outcomes were time from injury to onset of RE, incidence of tracheostomy, length of stay (in emergency medical center or hospital), ventilatory dependence at discharge, and in-hospital death.

### Statistical analysis

In the univariate analysis, all continuous variables were expressed as medians (interquartile ranges) and assessed using the Wilcoxon rank-sum test. Categorical variables were expressed as numbers and percentages and assessed using the two-tailed Fisher’s exact test. *P*-values < 0.05 were considered statistically significant. A multivariate analysis was performed using binomial logistic regression to determine the independent predictors of RE by calculating adjusted odds ratios (ORs) and 95% confidence intervals (CIs). In accordance with previous studies, we entered the following four variables, based on clinical plausibility and availability, into multivariate logistic regression analysis: “motor-complete injury” as a simple predictor based on initial evaluation of sacral sparing, “level of injury above C5” as evaluated by pre-admission neurological examination and MRI, “atelectasis” as a subjective post-admission factor that required radiographic interpretation by the physicians, and “CAS” as an objective post-admission factor mostly assessed by nursing staff at the bedside. However, we excluded ISS from the list of predictive variables for RE because of its difficulty to be clinically calculated promptly prior to admission. We also determined pneumonia in patients with CSCI as an ineligible predictor of RE because of its delayed onset (approximately 7 days) compared with atelectasis [1]. All statistical analyses were conducted using the JMP software (version 14.1; SAS Institute, Cary, NC, USA).

## Results

**Fig. 1:**
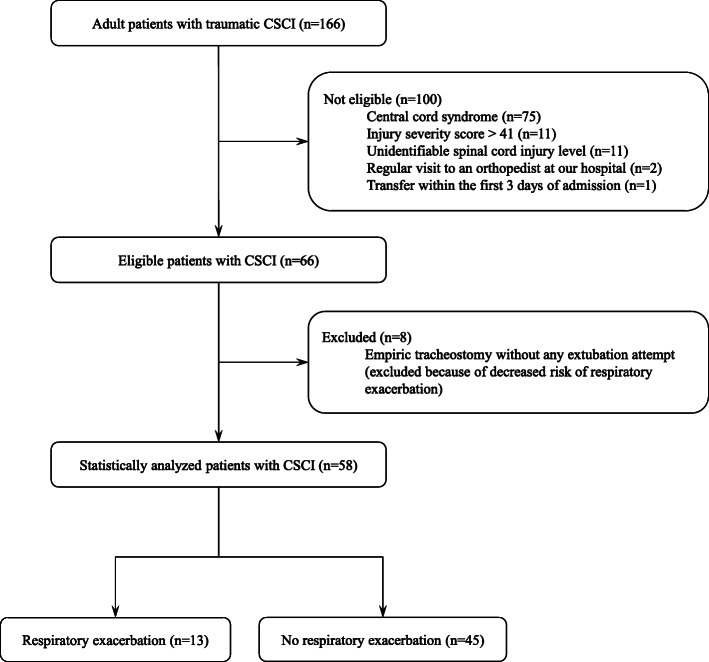
Case inclusion criteria and flow of patients in this analysis. CSCI, cervical spinal cord injury

During the 5-year study period, there were 33,899 ED visits at our hospital (19,015 by ground ambulance and 1784 by air ambulance). Our registry dataset included 1831 hospital admissions of the 8388 trauma victims who were under the primary care of emergency physicians. One thousand and thirty-five trauma victims had ISS > 16. The mean and median ISS were 17 ± 13 and 14 (interquartile range [IQR]: 9–25), respectively. Among the 166 consecutively enrolled patients diagnosed with traumatic CSCI, 66 were enrolled in the study. The Supplementary Figure shows the clinical trajectories of airway control in the 66 enrolled patients with CSCI, including eight who underwent empiric tracheostomy, to summarize the overall invasive airway management in this study. Of the 66 patients enrolled in this study, 43 (65.2%, 11 pre- and 32 post-admission) required intubation, including anesthetic intubation during standby surgery. Eleven patients were intubated pre-admission for the following causes: unstable airway due to acute traumatic retropharyngeal hematoma (*n* = 5), hypoxemia or hypercapnia associated with abdominal breathing (*n* = 4), and circulatory collapse with neurogenic shock (*n* = 2). The location where patients were intubated before admission were: ED (*n* = 7), former hospital (*n* = 3), and field by air ambulance doctor (*n* = 1). A total of 14/43 (32.6%) patients underwent empirical tracheostomy without attempting any extubation, including 8 patients with pre-admission and 6 with post-admission intubation. We finally performed statistical analyses on the data of 58 patients, excluding the 8 patients who had a lower risk of RE resulting from empirical tracheostomy (Fig. 1). Complete injuries (AIS grade A) were present in nine patients (15.5%), and incomplete injuries (AIS grade B, C, and D) were found in 49 patients (84.5%). Of the 58 patients with CSCI, 35 (60.3%) required post-injury intubation (3 pre- and 32 post-admission), 8 (13.8%) required tracheostomy, and 1 (1.7%) died. RE was observed in 13 (22.4%) patients at an average of 3.5 days from injury. No apparent worsening of neurological outcomes due to emergency RE intubation was observed. No RE directly led to death. Of these 13 patients, 6 (46.2%) required unexpected urgent intubation (average of 2.7 days post-injury) and 7 (53.8%) were re-intubated urgently within 72 h after the planned extubation (on average 4.3 days post-injury). Among the 58 patients with CSCI, 27 (46.6%) had motor-complete injury (AIS A and B) and 22 had injuries above C5 (37.9%). Eleven of the 27 (40.7%) patients with motor-complete injury and five of the 22 (22.7%) patients with neurologic injury above C5 required emergency intubation at RE. Three of the eight patients with both risk factors (motor-complete injury above C5) required emergent RE intubation (37.5%). Three (23.1%) of the 13 CSCI patients intubated with RE had both motor-complete injury and injury level above C5 as the representative risk factors for intubation before admission.

### RE versus non-RE

Table 1 compares the baseline and injury characteristics between the RE group and the control group. This study population did not include neurological C1–C2 (highest cervical) and C7–C8 (lowest cervical) levels of injury. Among all the patients with CSCI, the mean age was 68.9 years (median: 70, IQR: 65–75); 46 male (79.3%) and 12 female (20.7%) patients were included, and the mean ISS was 18.8 (median: 17, IQR: 16–22). There were no intracranial injuries or cases of hemorrhagic/obstructive shock observed during the initial treatment in this study. No patients with RE had AIS grade D injury. In injury-related characteristics, RE patients had a higher severity of overall injury (median: 25 vs. 17, *P* = 0.0023), whereas the Abbreviated Injury Scale for the chest (1–5) and concomitant chest injury (lung injury, bony thorax injury, thoracic vertebral fracture) observed in the RE group were not significantly greater than those in the control group. None of the patients had a frail chest. The RE group also had a higher frequency of motor-complete injury than the control group (84.6% vs. 35.6%, *P* = 0.0034). However, there were no significant differences observed in other baseline characteristics and injury-related factors among the groups. The treatment and clinical outcomes of the 58 patients are shown in Table 2. Of the 13 RE patients, 1 (7.7%) died because of postoperative sepsis. The overall incidences of CAS, atelectasis, and pneumonia during the acute 3-day phase post-admission or within 24 h before RE were 32.7, 25.9, and 3.4%, respectively. Surgical stabilization was performed in 31 (53.4%) patients, including three urgent and 28 elective surgeries. The mean time from injury to surgery for all 31 patients undergoing surgery was 5.6 days (median: 5, IQR: 2–9), with no significant difference between the RE and control groups (median: 3 vs. 6 days, *P* = 0.2362). The relationship between cervical spine surgery and RE was not statistically significant in either group. The following clinical outcomes were significantly higher in the RE group than in the control group: initial ICU admission (2 vs. 1; *P* = 0.1230), CAS (10 vs. 9; *P* = 0.0003), atelectasis (8 vs. 7; *P* = 0.022), tracheostomy (7 vs. 1; *P* < 0.0001), length of emergency medical center stay (median [days], 36 vs. 18; *P* = 0.0009), and hospital stay (median [days] 57 vs. 21; *P* = 0.0004); discharge with dysphasia (7 vs. 4; *P* = 0.0012), and discharge with ventilatory dependence (1 vs. 0; *P* = 0.0489).

**Table 1:** Baseline and injury characteristics of the study population

	Overall (*n* = 58)	RE group (*n* = 13)	Control group (*n* = 45)	Unadjusted OR (95% CI)	*P*-value
Age, median [IQR], y	70 [65–75]	75 [68–80]	70 [64–74]		0.1759
> 65 y, n (%)	42 (72.4)	11 (84.6)	31 (68.8)	2.38 (0.48–12.72)	0.3183
Male sex, n (%)	46 (79.3)	12 (92.3)	34 (75.6)	0.25 (0.03–2.21)	0.2640
Body mass index, median [IQR]	22 [20–24]	22 [21–25]	21 [20–25]		0.0572
> 24, n (%)	13 (22.4)	3 (23.1)	10 (22.2)	1.05 (0.24–4.56)	1.0000
CCI, median [IQR]	0 [0–1]	1 [0–3]	0 [0–1]		0.1020
> 1, n (%)	11 (18.9)	5 (38.4)	6 (13.3)	4.06 (0.99–16.63)	0.1010
Pulmonary centrilobular emphysema, n (%)	14 (24.1)	3 (23.1)	11 (24.4)	0.92 (0.21–3.98)	1.0000
Cervical OPLL, n (%)	13 (22.4)	3 (23.1)	10 (22.2)	1.05 (0.24–4.56)	1.0000
DISH, n (%)	8 (13.8)	2 (15.4)	6 (13.3)	1.18 (0.21–6.69)	1.0000
Initial GCS, median [IQR]	15 [14–15]	14 [13–15]	15 [14–15]		0.0827
< 15, n (%)	20 (34.4)	7 (53.8)	13 (28.8)	2.87 (0.81–10.19)	0.1112
Initial bradycardia, n (%)	18 (31.1)	5 (38.5)	13 (28.9)	1.53 (0.42–5.58)	0.5156
Initial hypotension, n (%)	14 (24.1)	4 (30.8)	10 (22.2)	1.55 (0.39–6.13)	0.7138
Fall, n (%)	36 (62.1)	9 (69.2)	27 (60.0)	1.50 (0.40–5.61)	0.7475
Motor vehicle accident, n (%)	18 (31.1)	2 (15.4)	16 (35.6)	0.32 (0.06–1.67)	0.3068
ISS, median [IQR]	17 [16–22]	25 [18–26]	17 [16–18]		0.0023*
ISS > 25, n (%)	14 (24.1)	7 (53.8)	7 (15.5)	6.33 (1.63–24.57)	0.0088*
ISS > 16, n (%)	53 (91.3)	13 (100)	40 (88.9)	N/A	0.5773
Abbreviated Injury Scale for chest					
1, n (%)	1 (1.7)	0	1 (2.2)	N/A	1.0000
2, n (%)	2 (3.4)	1 (7.7)	1 (2.2)	3.66 (0.21–63.03)	0.4010
3, n (%)	2 (3.4)	0	2 (4.4)	N/A	1.0000
4, n (%)	1 (1.7)	0	1 (2.2)	N/A	1.0000
5	0	0	0		
Lung injury, n (%)	2 (3.4)	1 (7.7)	1 (2.2)	3.66 (0.21–63.03)	0.4010
Bony thorax injury, n (%)	1 (1.7)	0	1 (2.2)	N/A	1.0000
Thoracic vertebral fracture, n (%)	4 (7.0)	1 (7.7)	3 (6.7)	1.16 (0.11–12.26)	1.0000
AIS grade					
A, n (%)	9 (15.5)	4 (30.8)	5 (11.1)	3.55 ((0.79–15.84)	0.1025
B, n (%)	18 (31.1)	7 (53.9)	11 (24.4)	3.61 (0.99–13.03)	0.0851
C, n (%)	17 (29.3)	2 (15.4)	15 (33.3)	0.36 (0.07–1.85)	0.3068
D, n (%)	14 (24.1)	0	14 (31.1)	N/A	0.0269*
Motor-complete injury, n (%)	27 (46.6)	11 (84.6)	16 (35.6)	9.96 (1.96–50.65)	0.0034*
Neurological CSCI level					
C3, n (%)	12 (20.7)	1 (7.7)	11 (24.4)	0.25 (0.03–2.21)	0.2640
C4, n (%)	10 (17.2)	4 (30.8)	6 (13.3)	2.88 (0.67–12.41)	0.2083
C5, n (%)	22 (37.9)	7 (53.9)	15 (33.3)	2.33 (0.66–8.17)	0.2078
C6, n (%)	14 (24.1)	1 (7.7)	13 (28.9)	0.21 (0.02–1.74)	0.1553
Level of injury above C5, n (%)	22 (37.9)	5 (38.5)	17 (37.8)	1.03 (0.28–3.66)	1.0000

*OR* odds ratio, *CI* confidence interval, *IQR* interquartile range, *RE* respiratory exacerbation, *CCI* Charlson Comorbidity Index, *OPLL* ossification of posterior longitudinal ligament, *DISH* diffuse idiopathic skeletal hyperostosis, *GCS* Glasgow Coma Scale, *ISS* Injury Severity Score, *ASIA* American Spinal Injury Association, *AIS* ASIA Impairment Scale, *CSCI* cervical spinal cord injury

*Statistically significant at *P* < 0.05

**Table 2:** Treatment and clinical outcomes of the 58 analyzed patients with CSCI

	Overall (*n* = 58)	RE group (*n* = 13)	Control group (*n* = 45)	Unadjusted OR (95% CI)	*P*-value
Intubation before admission, n (%)	3 (5.2)	2 (15.4)	1 (2.2)	8.0 (0.66–96.4)	0.1230
Initial ICU admission, n (%)	3 (5.2)	2 (15.4)	1 (2.2)	8.0 (0.66–96.4)	0.1230
CAS in acute stage, n (%)	19 (32.7)	10 (76.9)	9 (20.0)	13.33 (3.02–58.7)	0.0003*
Atelectasis in acute stage, n (%)	15 (25.9)	8 (61.6)	7 (15.6)	8.68 (2.12–34.4)	0.0022*
Pneumonia in acute stage, n (%)	2 (3.4)	1 (7.7)	1 (2.2)	3.66 (0.21–63.03)	0.4010
Steroid administration, n (%)	31 (53.4)	8 (61.6)	23 (51.1)	1.53 (0.43–5.40)	0.5461
Halo-Vest immobilization, n (%)	9 (15.5)	4 (30.8)	5 (11.1)	3.55 (0.79–15.94)	0.1025
Cervical spine surgery, n (%)	31 (53.4)	9 (69.2)	22 (48.9)	2.35 (0.63–8.76)	0.2248
Time from injury to surgery, median [IQR], days	5 [2–9]	3 [2.5–5]	6 [1–10]		0.2362
Tracheostomy, n (%)	8 (13.8)	7 (53.9)	1 (2.2)	51.33 (5.34–493.04)	< 0.0001*
Emergency medical center stay, median [IQR], days	21 [15–34]	36 [28–44]	18 [12–28]		0.0009*
Hospital stay, median [IQR], days	24 [15–54]	57 [35–62]	21 [13–36]		0.0004*
> 28 days, n (%)	24 (41.3)	11 (84.6)	13 (28.8)	13.53 (2.62–69.71)	0.0008*
Discharged with dysphasia, n (%)	11 (18.9)	7 (53.9)	4 (8.9)	11.95 (2.67–53.4)	0.0012*
Discharged with ventilator dependence, n (%)	2 (3.4)	2 (15.4)	0	N/A	0.0489*
In-hospital death, n (%)	1 (1.7)	1 (7.7)	0	N/A	0.2241

*CSCI* cervical spinal cord injury, *OR* odds ratio, *CI* confidence interval, *ICU* intensive care unit, *CAS* copious airway secretion

*Statistically significant at *P* < 0.05

### Predictors of RE

We conducted a multivariate analysis to identify plausible and available predictors for RE in clinical practice (Table 3). Our analysis revealed that CAS was an independent predictor for RE (adjusted OR: 7.19, 95% CI: 1.48–42.72, *P* = 0.0144).

**Table 3:** Multiple logistic regression analysis of independent risk factors for RE in patients with traumatic CSCI

	Odds ratio (95% confidence interval)	*P*-value
Complete motor injury	4.65 (0.73–40.52)	0.1036
Level of injury above C5	2.09 (0.39–12.48)	0.3851
Atelectasis	2.91 (0.53–16.48)	0.2144
Copious airway secretion	7.19 (1.48–42.72)	0.0144*

*RE* respiratory exacerbation, *CSCI* cervical spinal cord injury

*Statistically significant at *P* < 0.05

## Discussion

Here, we showed that CAS was a simple independent predictor for RE, especially during the acute 3-day phase. We suggest a reconsideration of early routine intubations based on neurological examinations in the ED for patients with CSCI.

The need for intubation for post-admission emergency RE in patients with CSCI decreased during the study period at our ED as compared with a similar setting nearly 20 years ago, when routine intubation based on the initial neurological assessment was not performed. In a 2003 retrospective study leading to the standardization of early routine CSCI intubation based on neurological assessment, the overwhelming majority of patients with complete quadriplegia, level of neurologic injury above C5, and complete quadriplegia above C5 required intubation (90, 87.5 and 95%, respectively) [3]. In a later study conducted in 2008, 97 of the 127 patients (76%) with low CSCI (C5–T1) and motor-complete injury were intubated for RE [23]. In the present study, intubation for RE in motor-complete injury, injury above C5, and motor-complete injury above C5 was performed in 3/15 (20.0%), 5/22 (22.7%), and 3/8 (37.5%) patients, respectively. Thus, in clinical practice, the occurrence of RE (emergency intubation after admission) has actually decreased over time, even without routine intubation before admission for motor-complete injury or CSCI above C5, which can be considered a benefit of multidisciplinary CSCI management improvement. With current respiratory management strategy as a premise, it is time to review the classic early routine intubation indications based on initial neurological assessment.

With the growing paradigm shift of early tracheostomy in CSCI [14, 15], we also found that empirical tracheostomy and ventilatory dependence were reduced in our study without pre-admission routine intubation based on neurological assessment compared with previous studies. In this study, 32.5% of the patients with CSCI requiring intubation progressed directly to early tracheostomy without any extubation attempt (empiric tracheostomy). Although not generally comparable, a 2013 study by Kornblith et al. reported that in 344 patients with spinal cord injury, in which CSCI accounted for 64.5%, empiric tracheostomy was performed in 84.6% [12]. Compared with a previous 2011 study, the ventilator dependence of CSCI patients at discharge or transfer was also found to be lower than the results of the previous 10 years (3.4% vs. 35.2%) [24]. In a study of tracheostomy in spinal cord injury, tracheostomy (both early and late) was found to be an independent predictor of ventilator dependence [12]. Fortunately, with the development of medical technology and strategies, the respiratory condition of patients with CSCI is expected to improve with the help of various treatments and devices such as physical therapy, antibiotics, diaphragmatic pacemakers, and ventilators [4]. The absence of pre-admission routine intubation based on neurological assessment in this study may have limited the patients who truly required endotracheal intubation, thereby reducing empiric tracheostomy and the ensuing ventilator dependence. If early tracheostomy becomes routine in patients with CSCI, there will be more empiric tracheostomies that could have been originally extubated. In future discussions of the usefulness of tracheostomy (both early and late) for CSCI, it will be necessary to take into account the appropriateness of the indications for intubation so as not to increase the number of empiric tracheostomies.

Even if pre-admission routine intubation is not performed for motor-complete CSCI above C5, the strategy of waiting until planned intubation with CAS as the index may prevent RE and reduce intubation leading to unnecessary tracheostomy. Additionally, our findings demonstrate that post-admission RE occurs during the 3-day acute phase post-injury. About 20 years ago, Claxton et al. reported that pneumonia and copious sputum were independent predictors of the need for mechanical ventilation [8]. Jackson et al. reported that aspiration (CAS in our study) was the earliest respiratory complication in patients with spinal cord injury, averaging 4.5 days after injury, while pneumonia occurred much later, averaging 24.5 days after injury [1]. We limited the definition of acute pneumonia in patients with CSCI to onset within 3 days of injury, because this study aimed to prevent RE early. Therefore, the incidence of pneumonia in the acute post-injury period as it relates to RE prophylaxis in this study was low, and no significant difference was found between the two groups (Table 2). Among the various factors that contribute to respiratory complications, CAS is often associated with sputum obstruction and sudden desaturation or ventilation failure as an airway problem, making it an easy indicator for early prediction of emergency intubation in CSCI patients. In addition, monitoring and evaluation of CAS are clinically easier to perform at the bedside than neurological assessment. Selective planned intubation based on CAS during the acute 3-day post-injury period may provide effective airway management with prevention of RE. We believe that a review of the appropriate indications for CSCI intubation in this study will also optimize future research on early tracheostomy. However, our results do not suggest the abandonment of resuscitative intubation as standard care for cardiopulmonary adverse events or empiric tracheostomy for RE prophylaxis in patients with CSCI.

Our study was limited mainly by the small sample size owing to the rarity of this severe trauma and the single-center retrospective design. Consequently, the independent variables that could be assessed using the logistic regression model were also limited. Moreover, RE is an infrequent outcome for patients with CSCI in every hospital because of the widespread acceptance of the early routine intubation practice based on the neurological assessment. In addition, this study could not evaluate the efficacy and appropriate timing of tracheostomy, nor the merits and demerits of empiric tracheostomy in patients with CSCI. We believe that future prospective studies will be needed to redetermine the indications for CSCI intubation in line with modern respiratory management. This is important in order to accurately validate the role of early tracheostomy as an important component of recent clinical practice for spinal cord injury.

## Conclusion

Even without early routine intubation based on neurological assessment (e.g., motor-complete injury above C5) before admission in patients with CSCI, advances in multidisciplinary CSCI management have reduced the incidence of RE and ventilator dependence. Selective intubation after admission with CAS monitoring during the acute 3-day period post-injury may effectively prevent RE without increasing unnecessary empiric tracheostomies. A contemporary review of the indications for intubation as the beginning of all CSCI respiratory management is important to accurately assess the roles, indications, and effective methods of the subsequent mechanical ventilation, weaning, extubation, and tracheostomy.

## Supplementary Information

**Additional file 1 Supplementary Figure** Clinical trajectories of airway management among 66 enrolled patients with CSCI including those who underwent empiric tracheostomy. CSCI, cervical spinal cord injury; RE, respiratory exacerbation; CPA, cardiopulmonary arrest

## Data Availability

The datasets used and/or analyzed during the current study are available from the corresponding author on reasonable request.

## Abbreviations

CSCI: cervical spinal cord injury
ED: emergency department
RE: respiratory exacerbation
ICU: intensive care unit
VAP: ventilator-associated pneumonia
MRI: magnetic resonance imaging
ASIA: American spinal injury association
AIS: ASIA impairment scale
ISS: injury severity score
CT: computed tomography
CAS: copious airway secretion
OR: odds ratio
CI: confidence interval
IQR: interquartile range
