# Supplementary material for: Timely intubation with early prediction of respiratory exacerbation in acute traumatic cervical spinal cord injury
Source: BMC Emerg Med. 2021 Nov 13;21:136. doi: 10.1186/s12873-021-00530-3 (PMC8590122; doi:10.1186/s12873-021-00530-3)
Supplement: Supplementary file 1 — Additional file 1 Supplementary Figure Clinical trajectories of airway management among 66 enrolled patients with CSCI including those who underwent empiric tracheostomy. CSCI, cervical spinal cord injury; RE, respiratory exacerbation; CPA, cardiopulmonary arrest [file 12873_2021_530_MOESM1_ESM.pdf]

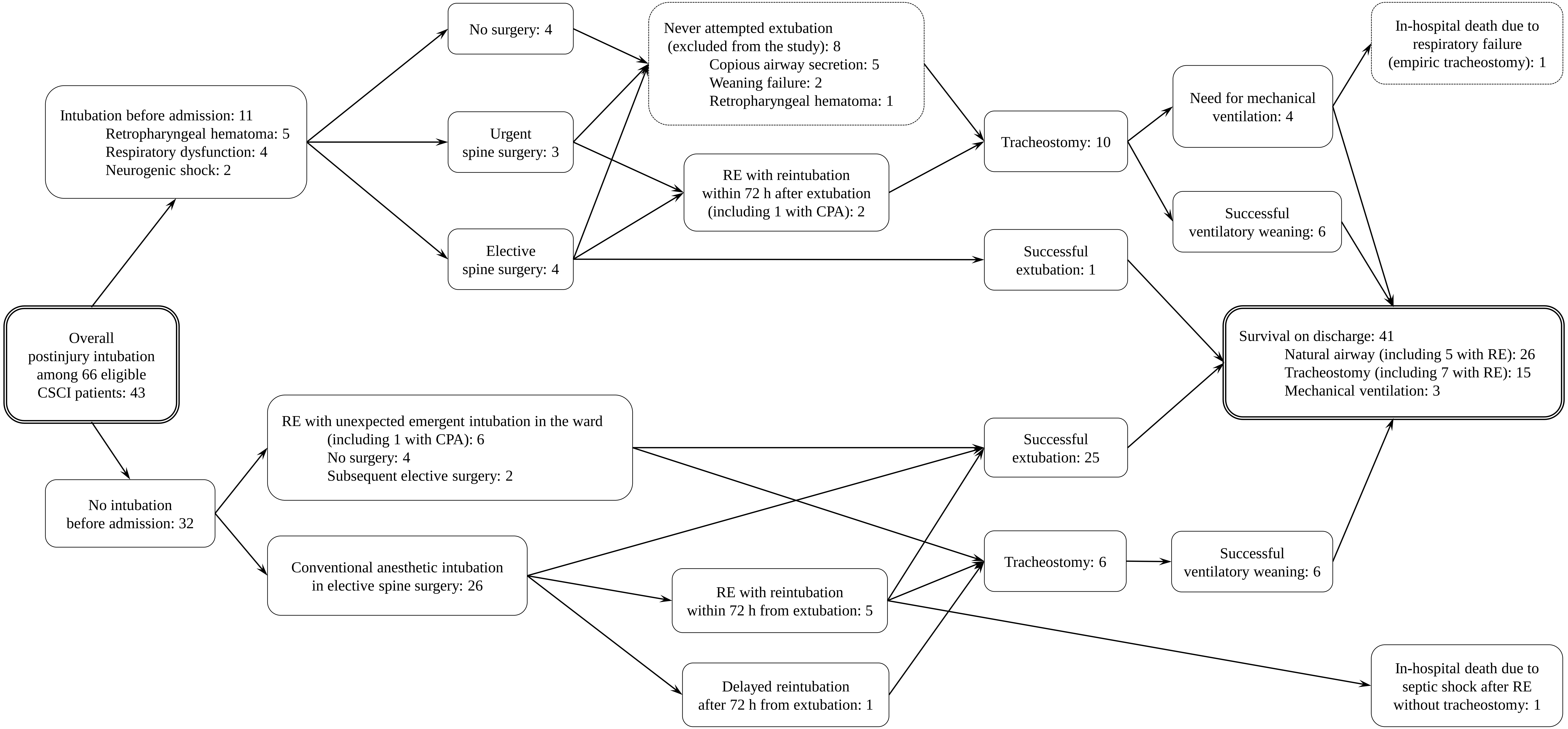

Supplementary Figure. Clinical trajectories of airway management among 66 enrolled patients with CSCI including those who underwent empiric tracheostomy. CPA, cardiopulmonary arrest; CSCI, cervical spinal cord injury; RE, respiratory exacerbation
